# Mission-driven e-professionalism in the medical field: shaping digital identity and virtual engagement

**DOI:** 10.3389/fmed.2024.1276839

**Published:** 2024-03-22

**Authors:** Shaista Salman Guraya, Fiza Rashid-Doubell, Denis W. Harkin, Salman Yousuf Guraya

**Affiliations:** ^1^Health Professions Education, Institute of Learning, Mohammad Bin Rashid University of Medicine and Health Sciences, Dubai, United Arab Emirates; ^2^Retired, Edinburgh, United Kingdom; ^3^Faculty of Medicine Health Sciences, Royal College of Surgeons in Ireland, Dublin, Ireland; ^4^Clinical Sciences Department, College of Medicine, University of Sharjah, Sharjah, United Arab Emirates

**Keywords:** e-professionalism, mission, digital identity, social networking sites, digital agency

## Introduction

The notion of “e-professionalism” means “*a distinct new paradigm requiring training, codes, and practices”* (1), a definition that differs from conventional professionalism in three distinct ways: first, the contextual variance of the digital context from the *real world*; second, the existing practices of social networking sites (SNSs) that affect a specific style of involvement, diagnostics, or adherence that are not solely under human control; and finally, the peculiar affordance of SNSs where generated content yields a heterogeneous configuration and reconfiguration of human engagement. In general, medical professionalism is considered a social contract between medicine and society and is perceived differently in different societies (2–7). This is further complicated when we consider virtual socialization, where issues are no longer contained by physical boundaries and can be disseminated by a mere touch. Professionalism in this realm appears more perplexing and harder to understand and practice. The construction of digital identity by the innocent sharing of online images, videos, and text narratives of a doctor-patient interaction by a medical student regarding workplace experiences has the potential to compromise the fundamental ethical principles of medical professionalism (8). Still in this open, chaotic, instant, and borderless socialization, medical ethos needs to be maintained and grounded in theory. Hence, the rapidly increased usage of SNSs in medical education demands training, and policies for a regulated application of e-professionalism remain rudimentary (9). Healthcare professionals (HCPs) should be made aware of the myriad possibilities of practices while interacting within SNSs and generating new ways of enactment to safeguard societal contracts (10). To understand the evolving attributes of e-professionalism, we have eluded the concepts of digital natives, digital ecologies and economies, digital competence, and digital agency. These concepts are then gelled into the emerging practice of mission-based e-professionalism in an attempt to embrace the transformation of conventional medical professionalism into the digital realm.

## Digital natives and digital identity

The present generations are digital natives, constituting highly qualified personnel in cyberspace with maturing cognition. Currently, online citizenship is permanent, uninterrupted, and uninhibited. However, the mechanisms, correspondence, communication, and publications of digital content are not well regulated in the absence of proviso. Thus, there are risks attached to the “openness” and connectivity of SNSs. The understanding of professionalism that relates to contextual sensitivity, relativity, and the use of judgment highlights a significant relationship between external behaviors, social expectations, and individual values. Thus, e-professionalism has heightened the substantial role of teaching future HCPs to “*craft and control”* digital identity (8, 11).

## Digital “*ecologies*” and “*economies*” of practice

Understanding the SNSs' impulsive, uninhibited, and immersive nature, a certain degree of control needs to be reflected in the professionals' behaviors. We understand that a successful modern practitioner is an expert at negotiating personal and professional identities while conserving the “*ecologies”* and “*economies”* of practices. The inability of digital natives to be digitally professional despite excellent technical knowledge is well known (12). This flashpoint reveals that it is not the lack of knowledge or power of technology but the specific mode of practice inside the technological realm (13). HCPs should be made aware of the possibilities of safe and ethical practices while interacting with SNSs and understand when boundaries of professionalism are being crossed. This shifts the focus from “*practitioner-in-isolation”* to “*practitioner-in-relation”* where mere regulation of professional behaviors will not suffice. Humanistic qualities, with observable behaviors and socialization conducted in the digital context, warrant a holistic appreciation to achieve “*digitally fit”* HCPs. This can be accomplished by educating and training all stakeholders, including medical and health students, faculty, HCPs, and patients, with a passion for working in digital harmony, which can safeguard professional values and behaviors in the medical field.

## Are digital natives digitally competent?

Goffman's notion of context collapse demonstrates a notable phenomenon where an individual in a digital context attempts to fulfill the expectations of a versatile audience. Potential consequences of this phenomenon include compromised online communications hampering effective communication, generational points of view leading to socially undesirable enactments, and misreckoning by one or more parties (14, 15). This concept has further stratified the idea of context collapse into “*collusions and collisions*” with the vital distinction of intention. The ubiquity of Internet access, along with the contingent intermingling of virtual spaces, has confirmed that SNSs are not fixed, nor does it determine the emergent phenomenon of human and non-human interactions, where capacity seems to be shared, yet an autonomous vacuum prevails. This “*shared capacity*,” where humans and non-humans interpret digital content from a pre-determined perspective, leads to “*knowing-in-practice”* where personal and professional practices lead to the enactment of performative human and material interactions (12). This formation of an autonomous vacuum, providing invisibility and anonymity, can challenge self-efficacy and perceived control, thus giving rise to the concepts of “*technologies-in-practice”* and “*affordances-in-practice.”* These attributes can then be conveniently applied for digital visibility, searchability, persistence of content, replicability, and shareability (16). Young HCPs, although called “*digital natives,”* are helpless when faced with permanency, mixing, interpolation, and shaping of digital content. However, the ability of an individual's agency (self vs. relational) could provide purpose in circumventing architectural affordances during the networked era (17).

## Idea of agency in the digital world

One might argue that the concept of legitimate compromise exists in conventional professionalism; however, it is not detrimental to the idea of agency. Rather, it represents change at a societal-institutional level as per Hodges discourse analysis, where decisions are expert-driven, high-tech, high-cost interventions balancing the individual against societal justice, as witnessed during the pandemic (18). Throughout the discourse on conventional professionalism, there remains a responsible individual for making ethical decisions using a specialized skill set and superior knowledge to align with the collective commitment to society. However, the relational agency in the digital world was also narrated by Fenwick (19), who suggested that expertise and responsibilities rely on mutual negotiations of professional practice and that technological artifacts demand a special kind of professional responsibility in the digital world. Keeping the idea of relational agency in mind, digital identity construction can be shaped in a collaborative manner where a “human-material” approach gives a nascent dimension to medical professionalism. In the digital context, the terms “*negotiating identities, maintaining distance, and recognizing and minimizing risk*” have specific meanings related to online interactions and behaviors.

## Digital culturally fit

According to Lu's (20) idea of “*culturally fit*,” individuals manifest a smooth interaction with the social environment if equipped with the right values and behaviors. Hence, an HCP who is digitally culturally fit needs to grasp the subtleties of cultural context to grasp the ever-changing cultural norms and expectations (21). Subsequently, a lack of such attributes would impact the individual's wellbeing. A digitally culturally fit HCP demands an understanding of the contextual fluid hierarchical interfaces in the digital realm. Such a frame of reference in the digital context demands a gestalt, responsible for safeguarding our social contract imposed by the complex contradictory demands of the digital self. This reflective view has implications for craft and control phenomena, which again represent shared capacity, distributed agency, and inexplicable human–non-human interactions. This narrative strengthens our pluralist frame of reference in the digital context, asking for a “*body”* responsible for safeguarding our social contract imposed by the complex contradictory demands of the digital self. With the absence of role models, a distinct void in the digital world appears, indicating the inadequacy of a professional identity formation framework for the empowered individual who can act beyond conventional physical realms. In addition, a behavior-based framework, along with its observable behavioral manifestations, creates a complex mix of cognitive, attitudinal, and personality characteristics that become vacant in the setting of collapsing contexts. While a value-based framework linked to morality and rooted in culture seems incomplete when every tweet and post, assembled and recontextualized in a piecemeal manner, is processed by artificially intelligent software.

## Mission and “*principled conscience*”

As described in Kohlberg and Kramer's (22) six stages of moral development, mission supports the view that attributes of e-professionalism are likely to arise during complex digital interactions, thus encompassing social orientation paradigms. Our recent study has presented a new set of findings that emerged from the field of transpersonal psychology with the multi-dimensionality of the required attributes in a digitally professional individual in the form of the Medical Education e-Professionalism (MEeP) framework (10). In this study, the “*mission”* was synonymous with the “*principled conscience*,” where understanding social mutuality and a genuine interest in others' welfare is based on the universal principles of respect and the demands of individual conscience (22). However, long before the saturation of SNSs, Boucouvalas articulated the importance of mission as being “*the experience of being part of meaningful wholes and in harmony with superindividual units such as family, social group, culture and cosmic order”* (23). Therefore, to accelerate moral maturity, we need the concept of “*mission”* to connect the philosophical literature of character (e.g., conformative, benevolent, universalist, and integrity), which manifests as observable qualities (e.g., tolerant, powerful, and a good communicator) by individuals who are not only cognitively aware but emotionally connected with qualities of self (e.g., reflection, conscientiousness, self-direction, and actualization).

## Relational agency and understanding of mission

The idea of “*relational agency*” and “*mission*” yields a professional individual who understands the desired attributes along with mutual adjustments of virtues, philosophical doctrine and specialized knowledge of systemic, relational, and material aspects of professional responsibility. As we understand, “mission” harnesses the concept of “*cybercivility*” which denotes a positive and inclusive digital culture, ensuring that individuals uphold professional values and demonstrate professionalism even in virtual environments (24). This “*mission”* grants freedom to “*self* ” from unitarist rules and moral imperatives by bringing a concept of status that demands a situated and collective web of commitments to make legitimate compromises through balancing conflicting values and priorities.

## Recommendations

This review emphasizes a critical concern regarding the necessity for HCPs to embody a diverse range of attributes from various models of medical professionalism. There is an inadequacy of simplistic and binary classifications for complex human experiences in the digital context, emphasizing the need for a holistic understanding of humanistic qualities and observable behaviors within digital socialization. We recommend a nuanced approach to digital professionalism, acknowledging the complexities and dynamics of digital interactions. Due to the risks associated with the openness and connectivity of SNSs, there is a need for a pluralist approach to developing HCPs prepared for the digital realm. This will draw on insights from the MEeP framework and acknowledge the contextual variances in the digital context (12). Additionally, the article recommends values essential for discerning appropriate digital behaviors, emphasizing the importance of context-dependent judgment. Finally, due to the substantial value of self-awareness, emotional intelligence, and reflective practice in navigating the challenges of the digital world, this research recommends that medical educators equip learners with tools for conscientiousness and actualization in the digital realm.

## Final opinion

We summarize the modeling of digital identity and virtual engagement using mission-driven e-professionalism in the medical field with three distinct and recognizable elements: “*solidification”* of identities by an understanding of “*mission,”* “*digitally cultural fitness”* with an understanding of the contextually fluid hierarchical interfaces, and an understanding of “*shared agency”* leading to new “*knowing-in-practices”* where personal and professional practices are an enactment of performative human and material interactions. This understanding is an absolute requirement for early career HCPs to safeguard patients' interests and their trust in their profession.

## Author contributions

SSG: Conceptualization, Data curation, Formal analysis, Investigation, Methodology, Resources, Visualization, Writing – original draft, Writing – review & editing. FR-D: Conceptualization, Supervision, Validation, Writing – review & editing. DH: Conceptualization, Supervision, Validation, Writing – review & editing. SYG: Conceptualization, Supervision, Validation, Writing – review & editing.
